# Species- and Caste-Specific Gut Metabolomes in Fungus-Farming Termites

**DOI:** 10.3390/metabo11120839

**Published:** 2021-12-04

**Authors:** Nanna Hjort Vidkjær, Suzanne Schmidt, Haofu Hu, Kasun H. Bodawatta, Christine Beemelmanns, Michael Poulsen

**Affiliations:** 1 Section for Ecology and Evolution, Department of Biology, University of Copenhagen, Universitetsparken 15, 2100 Copenhagen, Denmark; Suzanne.Schmidt@bio.ku.dk (S.S.); Haofu.Hu@snm.ku.dk (H.H.); 2 Natural History Museum of Denmark, University of Copenhagen, Universitetsparken 15, 2100 Copenhagen, Denmark; Bodawatta@snm.ku.dk; 3 Leibniz Institute for Natural Product Research and Infection Biology e.V., Hans-Knöll-Institute (HKI), Beutenbergstraße 11a, 07745 Jena, Germany; Christine.Beemelmanns@hki-jena.de

**Keywords:** GNPS, LC-MS, LC-MS/MS, Macrotermitinae, *Macrotermes*, *Odontotermes*, symbiosis, *Termitomyces*, metabolomics, gut, MolDiscovery

## Abstract

Fungus-farming termites host gut microbial communities that contribute to the pre-digestion of plant biomass for manuring the fungal mutualist, and potentially to the production of defensive compounds that suppress antagonists. Termite colonies are characterized by complex division of labor and differences in diet between termite size (minor and major) and morphological (worker and soldier) castes, and this extends to the composition of their gut microbial communities. We hypothesized that gut metabolomes should mirror these differences and tested this through untargeted LC-MS/MS analyses of three South African species of fungus-farming termites. We found distinct metabolomes between species and across castes, especially between soldiers and workers. Primary metabolites dominate the metabolomes and the high number of overlapping features with the mutualistic fungus and plant material show distinct impacts of diet and the environment. The identification of a few bioactive compounds of likely microbial origin underlines the potential for compound discovery among the many unannotated features. Our untargeted approach provides a first glimpse into the complex gut metabolomes and our dereplication suggests the presence of bioactive compounds with potential defensive roles to be targeted in future studies.

## 1. Introduction

Symbioses often include complex assemblies of organisms that affect each other’s ecology and evolution, potentially coevolve, and often encompass the production of chemical compounds that mediate signaling, digestive, or defensive roles [1,2,3]. Among the most complex host–symbiont associations are those in digestive tracts. These associations may involve specific lineages of microbial symbionts, and they tend to be dominated by bacteria that convert dietary components and produce essential vitamins, cofactors, and short-chain fatty acids for hosts. Host associations with consistent assemblies of specific gut community members are consequently promising models to explore the consistency and identity of gut metabolites. Metabolomics is increasingly allowing insights into the chemical ecology of small molecules that mediate interactions within such complex symbioses [4,5].

Termites host long-term associations with diverse gut microbial communities that serve distinct roles [6,7]. The most prominent community differences are associated with the termite host diet and thus primarily differ between soil-, wood-, and fungus-feeding termites [8,9]. The fungus-farming termite sub-family Macrotermitinae has engaged in a co-evolved obligate symbiosis with species in the fungal genus *Termitomyces* (Agaricales: Lyophyllaceae) for ca. 30 MY. The adoption of a primarily fungal diet remodeled farming termite gut bacterial community compositions [9,10,11], shifting the prominent role of plant biomass degradation from the gut to *Termitomyces*. Consequently, fungus-farming termite gut bacteria encode fewer lignocellulose-degrading enzymes, but are enriched in enzymes targeting the fungal cell wall [9].

**Figure 1:**
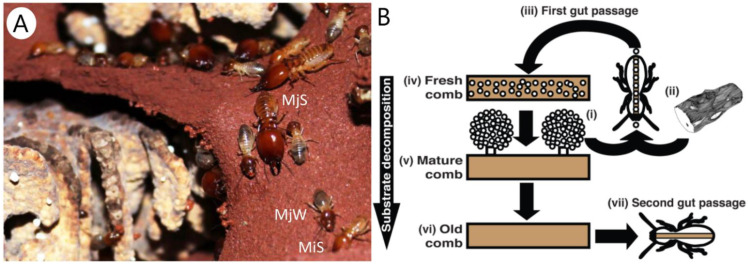
Fungus-farming termites. (**A**) Minor (not visible) and major (MjW) workers and minor (MiS) and major (MjS) soldiers of *Macrotermes natalensis* termites on soil terraces on which they maintain their fungus garden (Photo: Saria Otani). (**B**) Plant biomass processing in fungus-farming termites positions the termite gut centrally in both seeding combs and in termite nutrition. Asexual spores produced in nodules of *Termitomyces* (i) are mixed with plant biomass (ii) in a first gut passage (iii) that serves to seed the substrate with the fungal mutualist as it is deposited as fresh comb in the fungus garden (iv). *Termitomyces* in the mature part of the comb (v) produces more nodules, and after the plant substrate is fully utilized ((vi), old comb), fungal biomass is consumed in a second gut passage (vii) (reproduced from Poulsen et al., 2014 [15]).

Gut microbiomes are consistent within fungus-farming termite species, even across colonies located hundreds of kilometers apart [12]. Within species, quantitative gut community composition differences mirror primarily the division of labor between workers and soldiers (physical castes) but also sizes (minor and major), and ages (young and old) (Figure 1A) [12]. While soldiers engage in physical defense of the colonies, older workers forage for plant biomass that is brought back to the nest and ingested by younger workers along with asexual spores of *Termitomyces* in a fast first gut passage [13] (Figure 1B). These workers deposit this “primary feces” mix as fresh comb, on which *Termitomyces* grows to degrade carbohydrates (Figure 1B). As the comb matures, new asexual spores of *Termitomyces* are produced within so-called nodules, and older workers eventually consume the old comb [13,14] (Figure 1B). In contrast, termite soldiers rely on being fed by workers, and their guts do not appear to contain plant material but are rich in fungal mycelium [12].

Fungus-farming termites appear remarkably effective at preventing diseases, despite workers being exposed to pathogens and competitors when foraging [16]. Several mechanisms likely contribute to robust defense [17,18,19,20,21,22], and sanitation of plant material during the first gut passage has been proposed to play a role [23,24]. The presence of a complex gut microbiome implies the potential for bacterial production of bioactive defensive compounds [25,26] that could inhibit antagonists before they enter the nest and proliferate.

Given the dietary differences between castes and the potential role of the gut bacteria in defense, we tested two hypotheses. First, we hypothesized that dietary differences would affect gut metabolome compositions, with primary differences being between termite species and between workers and soldiers within species, following microbiome patterns. Secondly, we hypothesized that if obligate gut passage of the foraged plant material suppresses antagonists (cf. Bos et al., 2020 [16]), we would find more bioactive components of microbial origin in worker guts. To test these hypotheses, we performed untargeted LC-MS/MS analyses of worker and soldier guts from three South African species of fungus-farming termites. We used public and licensed MS/MS libraries for putative identification of chemical features and confirm a subset of compounds using commercially available analytical standards.

## 2. Results

We collected termites from three *Macrotermes natalensis*, one *Odontotermes* cf. *badius*, and three colonies of the undescribed species *Odontotermes* sp. in South Africa in 2018 (Table 1). When possible, the termites were separated by caste into major and minor workers, and major and minor soldiers (in *Macrotermes*, while *Odontotermes* only has one soldier caste). Workers were further divided according to age (young or old). The dissected guts were pooled by caste and age category as indicated in Table S1. We tested several extraction protocols and methanol was found to be the best suited solvent for our dual-purpose targeting both potential defensive compounds and a general profiling of termite gut metabolomes. Chemical compounds were thus extracted through homogenization in methanol and characterized by UHPLC-QTOF-MS/MS in both positive (ESI+) and negative (ESI−) ionization modes. We explored species- and caste-related differences in gut metabolomes by a combination of partial least-squares discriminant analysis (PLS-DA) performed in MetaboAnalyst 5.0 [27,28] and permutational multivariate analysis of variance (PERMANOVA) conducted with the Adonis function in the R package vegan [29]. These analyses were performed separately for the ESI+ and ESI− datasets (Table S3). To evaluate which chemical features contributed the most to the difference between termite species and castes, we used the variable importance in projections (VIP) acquired from the PLS-DA and one-way analyses of variance (ANOVA) performed in MetaboAnalyst 5.0.

### 2.1. Chemical Features Detected in the Termite Guts

**Figure 2:**
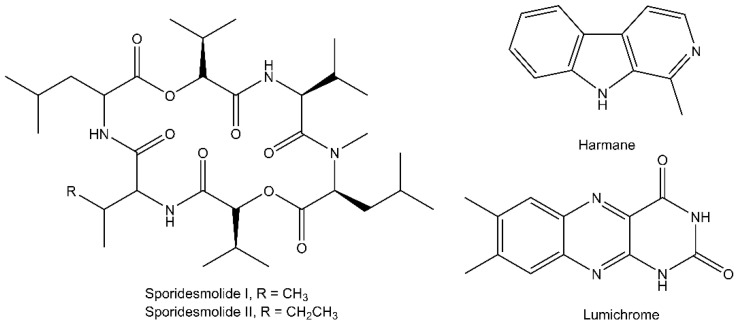
Compounds confirmed by analytical standards.

Processing the raw UHPLC-QTOF-MS/MS data with MZmine uncovered a total of 981 chemical features (418 from ESI− and 563 from ESI+) with unique retention times and *m*/*z* values (Table S2). The MZmine data was further analyzed using the Global Natural Product Social Molecular Networking (GNPS) platform [30,31,32]. Of the chemical features, 191 (94 from ESI− and 96 from ESI+) could be assigned to a chemical class based on our dereplication using public and licensed MS/MS libraries and subsequent propagation of compound annotations using the GNPS networks (Table S2). Complementary searches based solely on feature *m*/*z* values in custom compound databases encompassing compounds previously reported from the symbiosis and AntiBase 2017 provided several hits. Of these, we were only able to verify lumichrome, harmane and sporidesmolide I and II by comparisons to authentic standards (Figure 2 and Table S2). Limited commercial availability of pure analytical standard compounds prevented verifications of more feature identities. Unverified hits based only on *m*/*z* values were not used for the assignment of feature compound classes.

**Figure 3:**
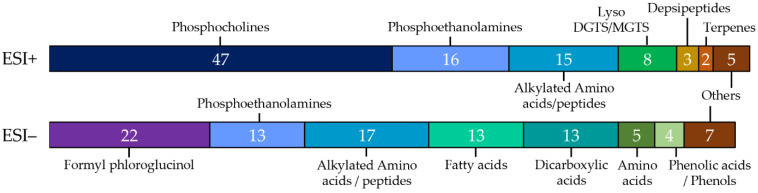
Compound classes of metabolome features. Distribution of the total of 191 chemical features that could be assigned to compound classes in ESI− (94 compounds) and ESI+ (96 compounds) based on our dereplication using public and licensed MS/MS libraries as well as subsequent propagation of the GNPS networks.

Across compound classes, phosphocholines, ethanolamines, fatty acids, alkylated amino acids/peptides and smaller organic acids (dicarboxylic acids and phenolic acids) dominated the gut extracts (Figure 3, Figures S1 and S2). Comparison with extracts of cultured *Termitomyces*, analyzed with the same methods and on the same instrument (Supplementary Material), revealed 74 overlapping features from ESI− and 59 from ESI+, corresponding to 18% and 11% respectively (Table S2). These features potentially originated from *Termitomyces* ingestion.

**Figure 4:**
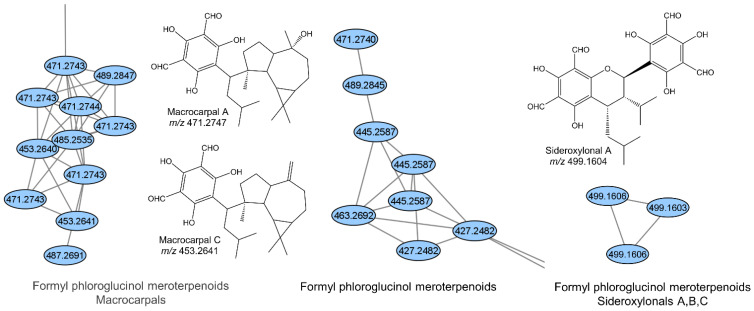
Formylated phloroglucinol compounds (generated in ChemDraw), and their respective small GNPS networks including the *m*/*z* values of [M - H]^−^.

The GNPS molecular networks provided visual representation of the gut chemical space (Figures S1–S5). The largest network (1) for the ESI− data contains two larger subnetworks representing alkylated amino acids/peptides and formyl phloroglucinol meroterpenoids (macrocarpals, Figure 4 and Supplementary Material), whereas the rest of this network remains unannotated. The second largest network (2) has several annotations of dicarboxylic acids and phenolic acids as well as a subnetwork representing additional formyl phloroglucinol meroterpenoids (Figure 4 and Supplementary Material). Apart from smaller networks representing phosphoethanolamines (network 3), fatty acids (subnetwork in networks 4 and 8), dicarboxylic acids (network 10) and sideroxylonals (network 21; Figure 4 and Supplementary Material), several minor networks and features represented as singles or doubles were left unassigned to a chemical class. These represent features of unknown chemical nature (Figure S1).

Our MS/MS library searches assigned several of the smaller ESI+ GNPS networks to phosphocholines (networks 6, 8, and 9), alkylated amino acids/peptides, also detected in ESI− (network 5) (Figures S6 and S7), and Lyso DGTS/MGTS (network 10). Network annotation coverage for the ESI+ data was, however, lower than the ESI− data, leaving the two largest networks (1 and 2) without annotations (Figure S2). To explore the ESI+ networks beyond MS/MS library annotations, we ran the ESI+ data through the in silico compound database search tool MolDiscovery [33], which is currently only implemented for ESI+ data in the GNPS environment. This provided an additional 93 putative feature annotations (Table S2 and Figure S5). MolDiscovery did not suggest identities for any features in ESI+ network 1, which mainly included features with little or no MS/MS fragmentation. This complicates further dereplication from the LC-MS/MS data alone. For network 2, MolDiscovery suggested three phosphocholines and a phosphoethanolamine (Table S2 and Figure S5). This network may thus represent another series of these compound classes, which would agree with the observed retention times and *m*/*z* values of its features (Table S2 and Figure S5). MolDiscovery also proposed additional compound classes for some of the smaller networks (Figure S5), including phosphoserines (networks 4 and 17), terpenes (network 21), and secosteroids/steroids (networks 12 and 26). These annotations appear plausible, as *Termitomyces* produces a variety of terpenes and steroids [34,35,36], and since phosphoserines are omnipresent as components of many proteins.

Given that MolDiscovery is an in silico tool, the annotations should be confirmed by orthogonal approaches, prior to more rigid biological interpretation. However, the limited commercial availability of analytical standards and the very small amounts of gut content material, precluding de novo structure elucidation, prevented verification of the MolDiscovery annotations. The results nonetheless demonstrate a clear potential for in silico data mining. Relevant candidate compounds with potential bioactivities were suggested by this approach, including alkaloids, terpenes, and peptides, which are promising targets for future compound discovery from this niche. Furthermore, for features where our MS/MS library searches also provided a dereplication hit, MolDiscovery annotations generally predicted the same compound class and did for instance predict several phosphocholines and phosphoethanolamines, and sporidesmolide I (Table S2). The identity of the latter compound was confirmed by an analytical standard.

### 2.2. Species-Level Differences in Termite Gut Metabolomes

**Figure 5:**
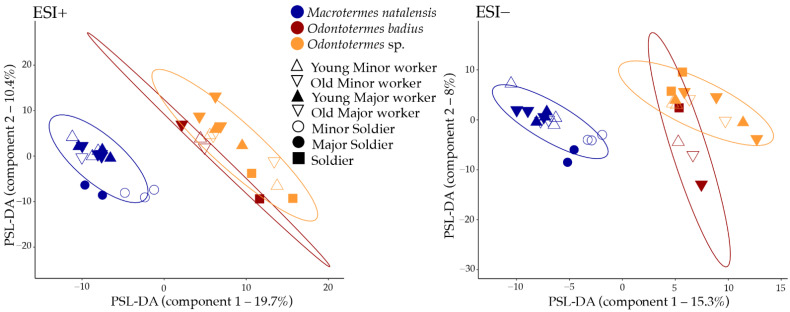
Metabolome similarities between gut samples. PLS-DA (partial least square discriminant analyses) plots showing chemical composition similarity of gut metabolomes between termite species and castes in the ESI+ and ESI− datasets, with 95% CI.

Our PLS-DA showed that termite species contributed the most to chemical composition differences, followed by within-species separation according to termite morphological caste (minor or major workers, minor, or major soldiers; Section 2.3). This pattern was observed in both the ESI+ and ESI− data sets (Figure 5, Table S4). The PERMANOVAs confirmed that chemical compositions were significantly different between termite species (ESI+: F = 3.192, R^2^ = 0.1857, *p* < 0.0001; ESI−: F = 3.351, R^2^ = 0.1931, *p* < 0.0001). Pairwise comparisons between species revealed that *M. natalensis* significantly differed from both *Odontotermes* species in both datasets (Table 2). We did not find significant differences between *Odontotermes* sp. and *O*. cf. *badius*, which may be due to the very small sample size of the latter collected from only one colony.

**Figure 6:**
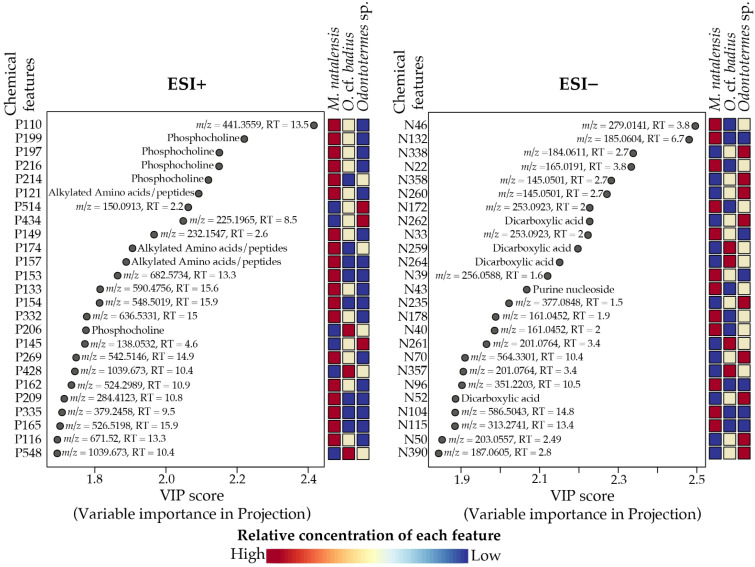
Top 25 chemical features contributing to the differentiation of gut metabolomes between three termite genera. Results based on the variable importance in projections (VIP) of the first component of the PLS-DA. Chemical features from the ESI+ dataset are given in the left panel and features from the ESI− dataset on the right. Features assigned to compound classes are given, while the *m*/*z* value and retention time are provided for unknown features. The heat maps show the relative abundance measured as mean peak areas across the individual castes for each VIP feature.

To evaluate which chemical features contributed the most to the differences between termite species, we used one-way ANOVA and the VIPs acquired from the PLS-DA (Figure 6 and Table S4). The majority of the 25 most contributing VIP features in the ESI+ dataset displayed the highest concentrations in *M. natalensis*. Eight of the features could be assigned to a compound class and these were dominated by phosphocholines and alkylated amino acids/peptides (Figure 6). In the ESI− dataset, only four of the 25 most contributing VIP features could be assigned to a chemical class, and all were dicarboxylic acids (pimelic acid, suberic acid, 3-methyladipic acid, 2-methylglutaruc acid; Table S4). Additional closely related dicarboxylic acids are likely present among the top 25 VIP features. The unknown features N260, N261, N357, and N358 display retention times and MS/MS fragmentation patterns comparable to dicarboxylic acid features N262 (3-methyladipic acid) and N52 (2-methylglutaric acid). These features are linked directly to the nodes N262 and/or N52 (edge cosine score 0.73–0.89) in the GNPS molecular networks (Figure S1) and the shifts in the *m*/*z* values of the unidentified features agree with simple modifications of the carbon backbone by one or three CH_2_ groups (Δ *m*/*z* 14 or 42, Figure 6). The likely presence of additional dicarboxylic acids among the VIP features merely emphasizes the importance of dicarboxylic acids as being discriminatory between guts from different termite species.

The one-way ANOVAs revealed 122 ESI+ and 90 ESI− features that were significantly differentially abundant between termite species (Table S4). The top 25 most important features from the PLS-DA displayed in Figure 6 were among the significant features from the one-way ANOVAs. Of the significantly different ESI− features, 32 were assigned to a chemical class and these were dominated by formylated phloroglucinol compounds (12), dicarboxylic acids (8), and phosphoethanolamines (4) (Table S4). The formyl phloroglucinol compounds were only found in the guts of termites from the single *O.* cf. *badius* colony and therefore they did not stand out in the PLS-DA. For the significantly different ESI+ features, 20 were assigned to the chemical classes, phosphocholines (11), alkylated amino acids/peptides (5), and phosphoethanolamines (4) (Table S4). MolDiscovery annotated an additional 16 features, including phosphoethanolamines (3), phosphoserines (2), diterpenoids (2), and piperidine alkaloids (2) (Table S4). The results of the one-way ANOVAs, thus supported the PLS-DA and emphasized the significance of dicarboxylic acids, phosphocholines, and phosphoethanolamines as important compound classes in the species differentiation of *Odontotermes* and *Macrotermes* termites.

A substantial portion of the features that significantly differentiated guts from different termite species were also detected in pure cultures of *Termitomyces* (16% in ESI+ and 27% in ESI−). This was particularly pronounced for the annotated features. Among the top 25 VIP features that were assigned to a compound class, most features were also detected in the *Termitomyces* extracts apart from the phosphocholines detected in ESI+ (Table S4).

### 2.3. Caste-Level Differences in Termite Gut Metabolomes

Due to the species-level differences, we investigated the significant differences between castes for each species separately in additional PLS-DAs and PERMANOVAs. For these comparisons, we excluded *O*. cf. *badius* due to the low sample size. We merged workers of different ages (young and old) and the two soldier castes in *M. natalensis* together as we did not see clear differences in their overall chemical compositions (Figure 5). We should note that we cannot rule out that more marked—and significant—differences could potentially have emerged with larger sample sizes, but the labor-intensiveness of sampling and dissecting guts prevented doing so in the current study. A substantial proportion of the features included in the species-level comparisons were only detected in one species, so these features were only included for the species in which they were detected in the caste-level comparisons. This meant that 167 ESI+ and 144 ESI− features were removed in *M. natalensis* and 189 ESI+ and 137 ESI− features were removed from *Odontotermes* sp.

The PLS-DA scores plot revealed a distinction between major and minor workers but not termites of different ages, nor between major and minor soldiers in *M. natalensis* (Figure S8 and Table S4). For *Odontotermes* spp. we did not find clear separations between major and minor workers. The caste separation was thus mainly driven by differences between workers and soldiers (Figure S8, Table S4). This applied to both termite genera and to data recorded in both positive and negative mode. The PERMANOVAs showed an overall significant effect of caste on the composition of *M. natalensis* gut metabolomes (Table 3). In the ESI+ dataset, the gut chemical composition of all castes (minor and major workers, and soldiers) differed significantly between each other, but the difference between minor and major workers was only marginally significant (Table 2). In the ESI− dataset, only chemical compositions in soldier guts differed significantly from minor and major workers. There was also a significant effect of caste in *Odontotermes* sp., but the post-hoc analyses revealed only marginally significant differences between major workers and soldiers in both ESI+ and ESI− datasets (Table 2). The lack of significance in pairwise tests in *Odontotermes* sp. is likely driven by the very small sample sizes per caste within this species.

**Figure 7:**
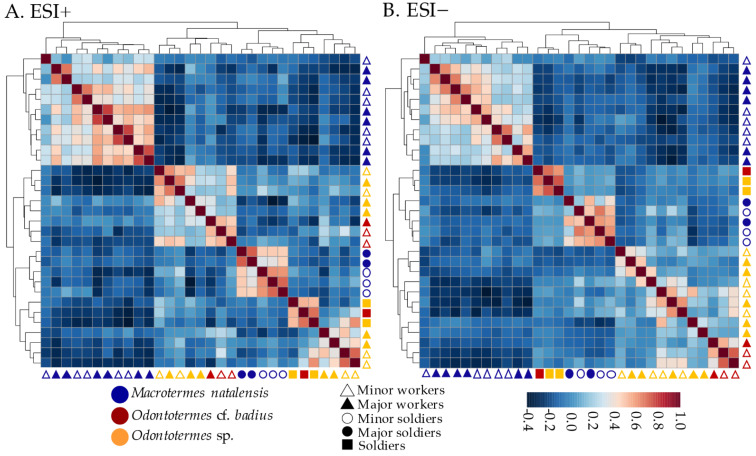
Pearson’s correlations between gut chemical communities of the termite species and castes. (**A**): Visualization of data recorded in positive ESI mode. (**B**): Visualization of data recorded in negative ESI mode. Positive correlations are depicted in red and negative correlations in blue. Dendrograms represent the similarity between chemical communities based on Euclidean distances.

To further investigate similarities between gut metabolomes of different castes, we conducted Pearson’s correlation analyses between the chemical communities in MetaboAnalyst [27,28] (Figure 7). These results complemented the patterns observed in the PLS-DA, demonstrating stronger correlations between castes within than between termite species for both ESI+ and ESI− datasets. However, in the ESI+ dataset, *Odontotermes* spp. workers generated two distinct clusters, where four worker samples were more similar in gut chemical composition to *Odontotermes* spp. soldiers than to congeneric workers (Figure 7). Soldiers correlated strongly within termite species in both datasets, and soldiers from all species clustered together (Figure 7).

**Figure 8:**
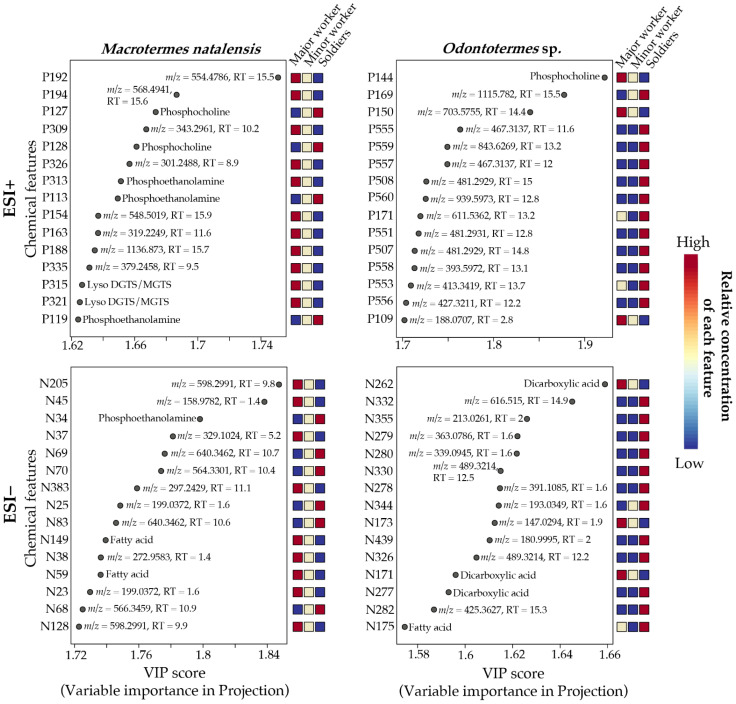
Top 15 chemical features contributing to the differentiation of gut metabolomes between termite castes (soldiers, major, and minor workers) of *M. natalensis* and *Odontotermes* spp. Results are based on the variable importance in projections (VIP) of component 1 of the PLS-DA. To obtain these, PLS-DAs were conducted separately for each of the termite genera (Figure S8). Chemical features from the ESI+ dataset is given in the top two panels and features from the ESI− dataset in the bottom two panels. Features that were assigned to compound classes are given within the figure, while the *m*/*z* value and retention time in minutes are provided for unknown features. The heat maps show the relative abundance measured as mean peak areas across the individual castes for each VIP feature.

To evaluate which chemical features contributed the most to differentiating castes within species, we took the same PLS-DA and one-way ANOVA approach as for the species-level comparisons (Table S4). Figure 8 provides the 15 most contributing VIP features from the PLS-DAs (reduced to top 15 features due to lower VIP values for the caste comparisons). In *M. natalensis*, only three of these features could be assigned to a chemical class for the ESI− data, two fatty acids, and one phosphoethanolamine. Six VIP features in the ESI+ data could be assigned to a chemical class, including phosphoethanolamines (2), phosphocholines (2), and lyso DGTS/MGTS (2) (Figure 8). MolDiscovery additionally assigned two features as topostins (P192: topostin B-553 and P194: topostin B-567) (Table S4). In *Odontotermes* sp., four ESI− and one ESI+ of the top 15 VIP features were assigned to a chemical class. For ESI−, three features were dicarboxylic acids, and one was a fatty acid, whereas the single feature for ESI+ was a phosphocholine. For ESI+, MolDiscovery additionally suggested two sterols (P553 and P556) (Table S4).

The one-way ANOVAs revealed 80 ESI− and 139 ESI+ features for *M. natalensis* and 47 ESI− and 46 ESI+ features for *Odontotermes* sp. with significantly differentially abundant levels between castes. All top 15 VIP features from the PLS-DAs (Figure 8) were among the significant ANOVA features (Table S4). The dominant ANOVA features in *M. natalensis* were phosphocholines (20), lyso DGTS/MGTS (6), and phosphoethanolamines (4) detected in the ESI+ data (35 ANOVA features assigned to chemical class). Fatty acids (9) dominated in ESI− features, along with phosphoethanolamines (4) and dicarboxylic acids (3) (23 ANOVA features assigned to chemical class) (Table S4). In *Odontotermes* sp., four ANOVA features were assigned as phosphocholines. MolDiscovery further contributed five annotations, predominantly sterols (Table S4). Fatty acids (11), dicarboxylic acids (9), and phosphoethanolamines (4) were the main compound classes among the ESI− features (34 ANOVA features assigned to chemical class) (Table S4). For both *M. natalensis* and *Odontotermes* sp., most significant features from the ANOVAs represented differences between workers and soldiers, whereas fewer significant features were found between major and minor workers (*M. natalensis*: 22 ESI−/28 ESI+ and *Odontotermes* sp.: 2 ESI−/4 ESI+). For the features with significant differences between major and minor workers, differences were also found between workers and soldiers. Thus, no features displayed exclusive significant differences between the two worker castes for either of the termite genera. This supports the inferences from the PLS-DA, from which the main differentiations were also between workers and soldiers, with the top 15 VIP features being dominated by features with higher abundance in either major workers or soldiers (Figure 8).

As we observed for the species-level differences, many features that were significantly differentially abundant between guts from different castes were also found in *Termitomyces* extracts (*M. natalensis*: 35% ESI−/18% ESI+ and *Odontotermes* sp.: 38% ESI−/26% ESI+) (Table S4), suggesting that dietary differences between castes contributes to differential abundances. Many of the annotated features could be derived from *Termitomyces*, with only five of the top 15 VIP features annotated to a compound class being absent in *Termitomyces* extracts (Table S4). Two of these features were the lyso DGTS/MGTS and the other three belonged to different compound classes (Table S4).

## 3. Discussion

### 3.1. Termite Gut Metabolomes Differ by Species and Caste

The most marked differences in gut metabolomes were between termite species followed by soldiers and workers differentiating within species. As hypothesized, this fits the general pattern of dietary differences and follows the general trend observed for termite microbiome compositions [4,8]. As with microbiome structure, our findings also point to overall highly consistent metabolomes, despite the potential for variation induced by environmental input. Pearson correlations indicated that soldier metabolomes from the three species and two genera—albeit with limited sampling from the two *Odontotermes* species—were more like each other than those of workers of their respective colonies of origin. This suggests that the soldier castes of different termite species may share metabolites that are specific to their digestion or defensive roles. It also underlines the importance of caste-related diet differences as major drivers of gut chemical compositions. Elaborate conclusions based on the metabolomes are nevertheless challenging due to the many unannotated features driving caste and species separations. However, phosphoethanolamines, phosphocholines, fatty acids, and dicarboxylic acids appear to be major drivers of the differentiation of both termite species and castes (Figure 6 and Figure 8). A number of fatty acids and organic acids have been identified in *Termitomyces* [34,37], pointing to a dietary origin of these compounds. The omnipresence of phosphoethanolamines and phosphocholines precludes conclusions regarding their origins and roles.

### 3.2. Diet and Environment Influences Termite Gut Metabolomes

We observed a substantial overlap between the metabolomes of the termite gut and that of cultured *Termitomyces*, where many annotated features were also detected (Tables S2 and S4). Termite species are broadly associated with different clades of fungal symbionts, which may have different chemical compositions. Such differences may contribute to termite species and genus separations and be one of the reasons underlying the large number of features that were exclusively detected in one termite species. *Termitomyces* also seems to significantly contribute to metabolome variation across castes, particularly between workers that ingest plant biomass, nodules, and *Termitomyces* fungus comb, and soldiers only ingesting nodules fed to them by workers. Although fatty acids, terpenes, and steroids are major compound classes produced by *Termitomyces* [35,38,39], only fatty acids were among the dominating gut features. Sterols and terpenes are generally poorly detected with LC-ESI−MS and this is a likely explanation for their poor representation. Given that our results point to an impact of *Termitomyces* on gut metabolomes, terpenes and steroids should be better characterized by complimentary GC-MS analyses in future studies. Many of the gut features that we also detected in cultured *Termitomyces* extracts were not annotated, despite that 257 compounds have been reported from *Termitomyces* species [40]. This suggests that we have only scratched the surface of *Termitomyces* metabolite production. Nodule and fungus comb analyses across termite species should be included in the future to gain insights into the role of specific *Termitomyces* species and biomass ingested by specific colony members in driving gut metabolome compositions.

Gut microbial degradation of dietary components is well-established in mammals and other insects [41,42] and may also contribute to termite gut metabolomes. Enterolactone is a well-established gut microbial metabolite of plant lignans, which also occur in the predominantly woody material collected by termites [43]. Enterolactone presence (Table S2) therefore suggests similar microbial conversion in termites. We also detected 3-(3-hydroxyphenyl)propionic acid (Table S2), which is a known metabolite produced by gut microbes following flavonoid and hydroxycinnamic acid ingestion [41]. *Termitomyces* spp. produce both hydroxycinnamic acids and flavonoids (e.g., caffeic acid and quercetin), which are also widespread in plant material [37,44,45]. The absence of these compounds in the guts may be the result of rapid microbial degradation and this is supported by the detection of 3-(3-hydroxyphenyl)propionic acid. The termite gut likely also contains multiple compounds formed by degradation of fungal cell walls, such as simple carbohydrates. These were not detected in our extracts, which may be due to our extraction protocol omitting more polar compounds. Fungal degradation products in the termite gut could be targeted in future analysis.

The formylated phloroglucinols (macrocarpals and sideroxylonals) (Figure 4) that were tentatively identified (Supplementary Material and Figure S9) in the guts of the *O.* cf. *badius* colony Od189 are predominantly found in *Eucalyptus* plants where more than 70 compounds are currently characterized across 39 species [46]. Eucalyptus is common in South Africa and seem prone to attacks by fungus-farming termites [47], making plant material the most plausible origin. Our detection of these compounds in termite guts therefore points to a clear environmental influence on gut chemical compositions. We only observed the formylated phloroglucinols in one colony and most abundantly in workers, with only a few being detected in soldiers (macrocarpal feature N268 and N310 and sideroxylonal feature N283) (Tables S2 and S3). This distribution further supports plant material origin, given that foraged plant material likely differs among colonies and is only ingested by workers. Formylated phloroglucinols often display great structural diversity with series of isomeric compounds usually co-occurring [46]. This diversity was reflected in gut extracts where three GNPS networks corresponding to at least three different structural classes, including macrocarpals and sideroxylonals, were tentatively identified (Figure 4; Supplementary Material). Without further data, it is however not possible to determine the exact nature of individual compounds. The formylated phloroglucinols have been in focus as diverse and interesting structural scaffolds and for their diverse bioactivities, which include antimicrobial properties. Whether plant-derived bioactive compounds play a role in termite pathogen defense remain unknown but could be evaluated in future studies.

### 3.3. Compounds with Reported Bioactivities

A recent review uncovered that a diverse range of chemical compounds has been elucidated from the fungus-farming termites, *Termitomyces*, and bacterial symbionts from guts and fungus combs [40]. Many of these compounds could be implicated in pathogen defenses, as several have antimicrobial properties, including against ecologically-relevant bacteria and fungi [21,48]. It has long been assumed that gut passage is instrumental to suppressing or eliminating incoming competitors or antagonists of *Termitomyces*. Bos et al. [16] suggested that this was unlikely to be a gut role, as diverse fungi that could challenge the symbiosis pass through the termite gut. Our analyses are consistent with this finding as we do not detect many antimicrobial compounds, nor do we find features that correspond to bioactive compounds previously reported from the symbiosis [40]. We should, however, note that many features remain unknown and that many natural products are poorly represented in MS/MS libraries. Further, our untargeted LC-MS approach does not offer universal detection of all compounds in complex extracts and especially lower concentration features may be missed. Finally, limited analytical standards and small amounts of gut material pose restrictions on further identification approaches, such as feature isolation for de novo structure elucidations. Despite these limitations, we verified interesting bioactive compounds of likely microbial origin, underlining the potential for compound discovery among the numerous unannotated features. The identities of these compounds were confirmed with commercially available analytical standards.

The cyclic hexadepsipeptides, sporidesmolide I and II, were consistently present in both termite genera (Table S2; Figure 2). Sporidesmolide I–V, originally isolated from cultures of *Pithomyces* [49], have previously been discovered in fungus combs of both *Macrotermes* and *Odontotermes* [22]. Limited work has thus far been done to uncover the bioactivities of these compounds. Other cyclic hexadepsipeptides have antimicrobial activity [50], but one study on the sporidesmolides found no activity against a variety of bacteria and fungi [51]. Within the sporidesmolide complex, I, II, and V are usually the dominating components with lower quantities of the more polar III and only traces of sporidesmolide IV. This pattern was also observed in fungus combs [22]. When the termites ingest the fungus comb, sporidesmolide I, II, and V may thus be consumed in substantially higher concentrations than the remaining sporidesmolides. Concentration differences could explain the absence of sporidesmolides with low *Termitomyces* abundance and the dominance of sporidesmolide I and II, but not the absence of sporidesmolide V. Differential gut degradation of individual sporidesmolides may however affect gut concentrations and explain why we do not detect sporidesmolide V.

The β-carboline alkaloid harmane was also detected in the gut extracts of both termite genera (Table S2; Figure 2) [52]. Previous reports indicate that harmane can be cytotoxic [53], stimulate the innate immune responses in nematodes [54] and has antimicrobial and antifungal activities [53,55,56]. We only detected harmane sporadically but most frequently in workers, with only a single occurrence in major soldiers in *M. natalensis* colony Mn187 (Tables S2 and S3). We hypothesize that harmane is of gut bacterial origin, similarly to the proposed producers in the guts of the closely related compound norharmane in *Reticulitermes* termites [57]. The inconsistent distribution of harmane may point to its production only being triggered under specific circumstances.

Lumichrome, a heterotricyclic compound, was observed in both termite genera (Tables S2 and S3; Figure 2), albeit inconsistently (Tables S2 and S3). Lumichrome is a natural light-sensitive metabolite of the naturally occurring riboflavin (vitamin B2), but it is also enzymatically produced by some bacteria [58,59]. Treatment with lumichrome has been shown to promote plant growth [60,61,62], linked as a natural inducer for larval metamorphosis in *Halocynthia roretzi* [63], and it activates the LasR quorum sensing receptor of *Pseudomonas aeruginosa* [64]. This suggests important regulatory roles across kingdoms, but its origin and potential effects in farming termite guts remain to be established.

The ecological roles of sporidesmolides, lumichrome, and harmane remain unknown, but they are likely all produced by symbionts. Our detection of these compounds underlines the potential for further compound discovery from the farming symbiosis. Future studies focusing on these and other bioactive compounds reported from the symbiosis [40], using targeted and more sensitive analytical approaches are thus warranted. When coupled with activity assays, this could provide valuable insights into their ecological relevance and the differences observed between termite castes and species. Additional defensive compounds could have been missed in our untargeted approach on healthy nests, as they may only appear in infected colonies. Several chemical compounds have been discovered from the symbiosis [40], and our work adds to the notion that we have yet to fully characterize the diversity and roles of chemical compounds mediating the complex symbiotic community.

Our metabolomics analysis provides a one-time snapshot of a complex gut environment, for which chemical analysis is challenging and restricted by small sample quantities limiting options for feature annotation and verification. We nonetheless identified key factors that appear to shape gut metabolomes including dietary and environmental contributions, but perhaps most importantly, we established clear consistencies within and between termite species and castes. Among the compounds we identified are known bioactive molecules and our dereplication uncovered additional potential targets for future work focusing on identifying compounds with potential implications in termite defenses.

## 4. Materials and Methods

### 4.1. Termite Collections and Gut Dissections

Three *Macrotermes natalensis* (Mn186, Mn187, and Mn190) and one *Odontotermes* cf. *badius* (Od189) colonies were excavated in South Africa in 2018 together with three colonies from an undescribed *Odontotermes* sp. species (Od191, 192, and Od194) (Table 1). When possible, major and minor workers, and major and minor soldiers (in the case of *Macrotermes*) were collected (Table S1). Major and minor workers were further divided according to age, estimated based on termite abdomen color [65]. Workers with a mostly white or light reddish-brown abdomen were designated as “young”, while workers having a dark brown to black abdomen were designated as “old”. We randomly selected 20 (*M. natalensis*) or 40 (*Odontotermes* spp.) individuals per caste and age category per technical replicate. Guts were dissected in sterile conditions using forceps and subsequently pooled. Three technical replicates were obtained in cases where the number of individuals allowed it (see Table S1 for details).

### 4.2. Chemical Extraction

Pooled guts were transferred to 2-mL screw cap reaction vials along with 1 mL of methanol and treated in a homogenizer with glass beads (0.25–0.50 mm) for 2 min. The samples were then centrifuged (5 min, 7300 rpm, 10 °C) and the supernatant transferred to a glass vial. The extraction procedure was repeated twice more for each sample (2 × 1 mL methanol), and the resulting supernatants combined and concentrated under reduced pressure to yield crude extracts. These were then dissolved (10% aqueous methanol, 1 mL) and fractionated by SPE (Solid Phase Extraction) (CHROMABOND HR-X, 30 mg, 1 mL), first washing with 10% aqueous methanol (3 × 1 mL) and then eluting with methanol (3 × 1 mL) and isopropanol (3 × 1 mL), with the methanol and isopropanol fractions collected in the same vials. SPE fractions were concentrated under reduced pressure and stored at −20 °C until UHPLC-QTOF-MS/MS analysis. Prior to analysis, samples were dissolved in methanol (100 µL) and centrifuged, before the supernatant was transferred to HPLC auto sampler vials.

### 4.3. Chemical Analysis

Samples were analyzed using an Agilent UHPLC-QTOF-MS/MS system, consisting of a 1290 Infinity UHPLC (Agilent Technologies, Torrance, CA, USA) equipped with a Poroshell 120 phenyl-hexyl column (250 × 2.1 mm, 2.7 µm particles). The column was eluted using a linear gradient consisting of A: HPLC-MS grade water + 20 mM of formic acid and B: HPLC-MS grade acetonitrile + 20 mM of formic acid. The gradient ran from 10–100% B over 15 min, followed by 100% B for 2 min, returning to 10% B over 0.1 min and equilibrated for 1.9 + 2.0 min (post run time) prior to the next injection. An injection volume of 1 µL was used. A constant flow of 0.35 mL/min was used, and the column maintained at 60 °C. This was coupled to an Agilent 6545 QTOF-MS equipped with an Agilent Dual Jet Stream electrospray ion source. The samples were analyzed in both negative and positive polarity by performing two separate injections. The ESI source parameters were: drying gas temperature, 250 °C; gas flow, 8 L/min; nebulizer gas pressure, 40 psig; sheath gas temperature, 300 °C; sheath gas flow, 12 L/min; capillary voltage, 4000 V; nozzle voltage, 500 V. Mass spectra were recorded at 10, 20, and 40 eV as centroid data for *m*/*z* 85–1700 in MS mode and *m*/*z* 30–1700 in MS/MS mode, with an acquisition rate of 10 spectra/s. Lock mass solution in 70:30 methanol:water was infused in the second sprayer using an extra LC pump at a flow of 15 μL/min using a 1:100 splitter. The solution contained 1 μM of tributylamine (Sigma–Aldrich, St. Louis, MO, USA) and 10 μM of Hexakis (2,2,3,3-tetrafluoropropoxy) phosphazene (Apollo Scientific Ltd., Cheshire, UK) as lock masses. The [M + H]^+^ ions (*m*/*z* 186.2216 and 922.0098 respectively) of both compounds was used.

Raw LC-MS/MS data were converted to the mzXML format using the ProteoWizard msconvert tool (https://www.reifycs.com/AbfConverter/index.html, accessed on 14 May 2021) before pre-processing in MZmine 2 v. 2.37 [30,31] (parameters included in the Supplementary Material). Data were first baseline corrected, followed by peak detection, isotopic peak grouping, peak alignment, filtering, and gap filling. Feature lists with each feature representing a unique *m*/*z* value and retention time were exported in .csv format and edited to remove features that were also detected in the solvent and/or SPE blanks. For GNPS analysis, an .mgf file feature table and metadata file were uploaded to Global Natural Product Social Molecular Networking (GNPS) [32], following the METABOLOMICS-SNETS-MZMINE workflow with the following parameters: precursor ion mass tolerance = 0.05 Da, fragment ion mass tolerance = 0.02 Da, min pairs cosine = 0.7, network TopK = 10, minimum matched fragment ions = 6, maximum connected component size = 100, Maximum shift between precursors = 500 Da. Several MS/MS libraries were used in the dereplication. The licensed NIST14 and the public MS/MS libraries, Public-Neg-VS15 and Public-Pos-VS15, which encompasses several public MS/MS libraries including MassBank, ReSpect, GNPS, Fiehn HILIC, MetaboBASE, Riken, and Fiehn/Vaniya Natural Product Library. The public libraries were downloaded from: http://prime.psc.riken.jp/compms/msdial/main.html#MSP (accessed on 14 May 2021). MS/MS library hits were used to further propagate the compound class annotations using the GNPS molecular networks. Custom-made databases of compounds previously identified from fungus-farming termites, bacteria, and *Termitomyces* [22,40] were also created and used in the dereplication. The custom-made databases were based on predicted *m*/*z* values (monoisotopic mass) and did not include MS/MS data. Further, the Antibase 2017 natural compound library was searched, but only contains mass spectrometry data for a very limited number of compounds meaning matches must be verified with complementary data. The newly developed in silico database search tool, MolDiscovery [33], currently implemented for ESI+ data only in GNPS, was used to search the ESI+ data using the following parameters: predefined database = AllDB (720.000 compounds), precursor ion mass tolerance = 0.01 Da, fragment ion mass tolerance = 0.01 Da, max charge = 2, minimum significant score = 30. Verifications of lumichrome, harmane, and sporidesmolide I and II with authentic analytical standards were done as previously described by Kildgaard et al., 2014 [52].

### 4.4. Statistical Analyses

For the statistical analyses, ESI+ and ESI− data were treated separately. Prior to data analyses features present in both the wash and elute fractions from the SPE purification were summed and then normalized to the weight of the individual pooled gut samples (Table S3). In cases with duplicate or triplicate sample pools collected from the same colony, the summed and weight normalized feature peak areas were averaged across colony replicates. These datasets were utilized to conduct statistical analyses in MetaboAnalyst 5.0 [27]. Missing values, i.e., features not detected in a given sample, were replaced by 1/5 of the minimum value of the corresponding feature, filtered by interquartile range to remove features with near constant values, log transformed and auto scaled. To identify the important chemical features driving differences between termite species and the castes within each species we conducted both partial least squares discriminant analyses (PLS-DA) [66] and one-way analyses of variance (ANOVA) with FDR adjusted *p*-values. In caste analyses, we excluded *O.* cf. *badius* due to the low sample sizes, with the exception that we added the *O.* cf. *badius* soldier sample (which was similar in composition; Figure 5) to *Odontotermes* sp. to obtain three replicates for the soldier caste in the test of *Odontotermes*. Using variable importance in projections (VIP) based on the PLS-DAs, we identified the most important chemical features driving the differences between different termite species and castes. We further investigated correlations between chemical communities of samples using Pearson’s correlations in MetaboAnalyst. Filtered, log-transformed, and auto-scaled data sets were exported from MetaboAnalyst and used to assess chemical community level differences between termite species and castes (based on Euclidian distances between communities) using permutational multivariate analysis of variance (PERMANOVA) tests with 10,000 permutations using the Adonis function in the Vegan package [29]. The wrapper package pairwiseAdonis was utilized to conduct pairwise comparisons between species and castes.

## Figures and Tables

**Table 1:** Colony identities and information. Colony codes, termite species, geographical location of collection, including GPS coordinates and date of excavation.

Colony ID	Termite Species	Location	GPS	Date Excavated
Mn186	*Macrotermes natalensis*	Experimental farm, Pretoria, South Africa	S25 44.600 E28 15.648	26 January 2018
Mn187	*Macrotermes natalensis*	Mookgophong, South Africa	S24 40.434 E28 48.275	29 January 2018
Mn190	*Macrotermes natalensis*	ARC, Pretoria, South Africa	ARC S24 40.512 E28 48.260	31 January 2018
Od189	*Odontotermes* cf. *badius*	ARC, Pretoria, South Africa	S25 43.903 E28 14.500	31 January 2018
Od191	*Odontotermes* sp.	Experimental farm, Pretoria, South Africa	S25 44.541 E28 15.400	2 February 2018
Od192	*Odontotermes* sp.	Experimental farm, Pretoria, South Africa	S25 44.554 E28 15.402	2 February 2018
Od194	*Odontotermes* sp.	Experimental farm, Pretoria, South Africa	S25 44.736 E28 15.761	5 February 2018

**Table 2:** Statistical analyses of pairwise differences in gut chemical profiles. Pairwise Adonis analyses between termite species (Mn = *M. natalensis*, Ob = *O.* cf *badius*, Os = *Odontotermes* sp.) and differences between castes (MjW = Major workers, MiW = Minor workers, S = Soldiers) of *M. natalensis* and *Odontotermes* sp. Significant effects (*p* < 0.05) are indicated with asterisks.

Variable	Comparison	F	R^2^	p_ajd_
**ESI+**				
Species	Mn vs. Ob	2.653	0.1284	0.0153 *
	Mn vs. Os	4.908	0.1641	0.0003 *
	Ob vs. Os	0.8697	0.0627	1.000
*M. natalensis* (castes)	MjW vs. MiW	1.672	0.1567	0.0402 *
	MjW vs. S	6.634	0.4243	0.0063 *
	MiW vs. S	4.915	0.3806	0.0237 *
*Odontotermes* spp. (castes)	MjW vs. MiW	1.112	0.1371	0.9197
	MjW vs. S	3.473	0.3666	0.0567
	MiW vs. S	2.386	0.3231	0.1714
**ESI−**				
Species	Mn vs. Ob	3.966	0.1805	0.0006 *
	Mn vs. Os	3.954	0.1366	0.0003 *
	Ob vs. Os	1.766	0.1196	0.1353
*M. natalensis* (castes)	MjW vs. MiW	1.529	0.1452	0.1902
	MjW vs. S	5.569	0.3823	0.0065 *
	MiW vs. S	4.145	0.3413	0.0279 *
*Odontotermes* spp. (castes)	MjW vs. MiW	0.9813	0.1229	1.000
	MjW vs. S	3.674	0.3798	0.0597
	MiW vs. S	3.853	0.4352	0.0857

**Table 3:** Statistical analyses of castes within species. Overall effects of caste on termite gut chemistry composition in *M. natalensis* and *Odontotermes* spp. in ESI+ and ESI− modes (PERMANOVA with 10,000 permutations). Significant effects (*p* < 0.05) are indicated with asterisks.

Dataset	Species	F	R^2^	*p*
ESI+	*M. natalensis*	4.470	0.4075	0.0002 *
	*Odontotermes* spp.	2.202	0.3285	0.0129 *
ESI−	*M. natalensis*	3.690	0.362	<0.0001 *
	*Odontotermes* spp.	2.556	0.362	0.0034 *

## Data Availability

The normalized LC-MS/MS peak area data used for statistical analyses are available as Table S3.

## Supplementary Materials

The following are available online at https://www.mdpi.com/article/10.3390/metabo11120839/s1, Figure S1: GNPS networks for ESI− showing annotated features, Figure S2: GNPS networks for ESI+ showing annotated features, Figure S3: GNPS networks for ESI− showing feature distribution between termite genera, Figure S4: GNPS networks for ESI+ showing feature distribution between termite genera, Figure S5: GNPS networks for ESI+ showing features annotated by MolDiscovery, Figure S6: ESI+ annotation of alkylated amino acids/peptides and GNPS network analyses, Figure S7: ESI− annotation of alkylated amino acids/peptides and GNPS network analyses, Figure S8: PLS-DA scores plots from the termite caste comparisons, Figure S9: GNPS sub-networks of formylated phloroglucinol compounds, Table S1: List of termite gut samples, Table S2: LC-MS/MS feature list, Table S3: Normalized LC-MS/MS peak areas, Table S4: Significant features from PLS-DAs and ANOVAs.
