# Supplementary material for: Species- and Caste-Specific Gut Metabolomes in Fungus-Farming Termites
Source: Metabolites. 2021 Dec 4;11(12):839. doi: 10.3390/metabo11120839 (PMC8707012; doi:10.3390/metabo11120839)
Supplement: Supplementary file 1 [file metabolites-11-00839-s001.zip › SuppInformation_doi 10.3390metabo11120839.pdf]

Supplementary Information

Species- and caste-specific gut metabolomes in fungus-farming termites

Termitomyces collection, cultivation, and chemical analysis

To examine if any of the features in gut metabolomes may originate from *Termitomyces*, we obtained fungal isolates from nine colonies of fungus-farming termites (IC0019, IC0020, IC0024, IC0036, IC0034, IC0035-1, IC0035-2, SA19-6, Micro) from Comoé National Park located in the Northeast of Ivory Coast. Nodules extracted from fungus comb taken from various termite nests were used to establish cultures in the lab at the University of Copenhagen. *Termitomyces* spp. were cultivated on Potato Dextrose Agar media or Yeast Malt Extract Agar (25 mL per plate, standard 15x90 mm) and stored at 25°C. After 28 days of growth, three *Termitomyces* plugs (each approximately 6.09 mm in diameter or 0.29 cm^2) were extracted using 3:1 ethyl acetate:isopropanol, with 1% formic acid and stored in -20°C. The samples were analysed using the same method used for analysing the termite guts.

Formylated phloroglucinol compounds tentatively identified in the guts of *Odontotermes* cf. *badius*

For the series of compounds tentatively identified as macrocarpals, a network of features with similar MS/MS fragmentation patterns, all having the diagnostic fragments *m*/*z* 250.08 and 207.02 were found among the GNPS networks (network 1 subnetwork – Figure S9). The *m*/*z* of several of these features corresponds to the closely related macrocarpals A, C, J, L and N (Figure S9) with a mass error of 1.9-2.3 ppm. Macrocarpals display great structural diversity with series of isomeric compounds often co-occurring (Dos Santos et al. 2019). Without further data, it is however not possible to determine the exact nature of individual compounds. In a subnetwork of GNPS network 2 (Figure S9) we uncovered a series of features sharing the same diagnostic fragmentation as the macrocarpals of network 1 but instead showing double loss of CO_2 from [M-H]-. These likely represent additional compounds with closely related structures. Further, a small GNPS network of three molecular features sharing the diagnostic fragments (*m/z* 181.01 and 249.07) of the related compound class, sideroxylonals, was also identified in the gut extracts with *m*/*z* values corresponding to sideroxylonals A, B and C (isomeric compounds, *m*/*z* 499.16, mass error 0.2-0.4 ppm) (Figure S9).

MZmine data processing parameters:

Baseline correction
Chromatogram type = BPC
MS level = 1
Use m/z bins = unchecked
Asymmetric baseline corrector
Smoothing = 100 000
Asymmetry = 0.01
Peak detection
Mass detector = centroid
Noise level 1.0E3 (MS1 detection) and 100 (MS2 detection)
CDF filename = unchecked
Chromatogram builder
Scans = MS level 1, Retention time 0-17
Min time span = 0.01
Min height = 3.0E3
Mass tolerance = 0.00 m/z or 20 ppm
Chromatogram deconvolution
Algorithm = baseline cut-off (min peak height = 4.0E4, peak duration range = 0.01-0.80 min, baseline level = 1.0E3)
m/z range for MS2 scan pairing (Da) = checked, 0.02
RT range for MS2 scan pairing (min) = checked, 0.2
Deconvolute co-eluting ions
m/z tolerance = 0.00 m/z or 20 ppm
Retention time tolerance = 0.2 min
Monotonic shape = unchecked
Maximum charge = 3
Representative isotope = Most intense
Align peaks
m/z tolerance = 0.00 m/z or 20 ppm
Weight for m/z = 75
RT tolerance = 0.2 min
Weight for RT = 25
Filter peaks
Minimum peaks in a row = unchecked
Minimum peaks in an isotope pattern = unchecked
Keep only peaks with MS2 scan (GNPS) = checked
Gap filling
Intensity tolerance = 10%
m/z tolerance = 0.00 m/z or 20 ppm
Retention time tolerance = 0.3 min
RT correction = Unchecked

References

Dos Santos, B.M., et al., *Quantification and Localization of Formylated Phloroglucinol Compounds (FPCs) in Eucalyptus Species.* Frontiers in plant science, 2019. 10: p. 186-186.

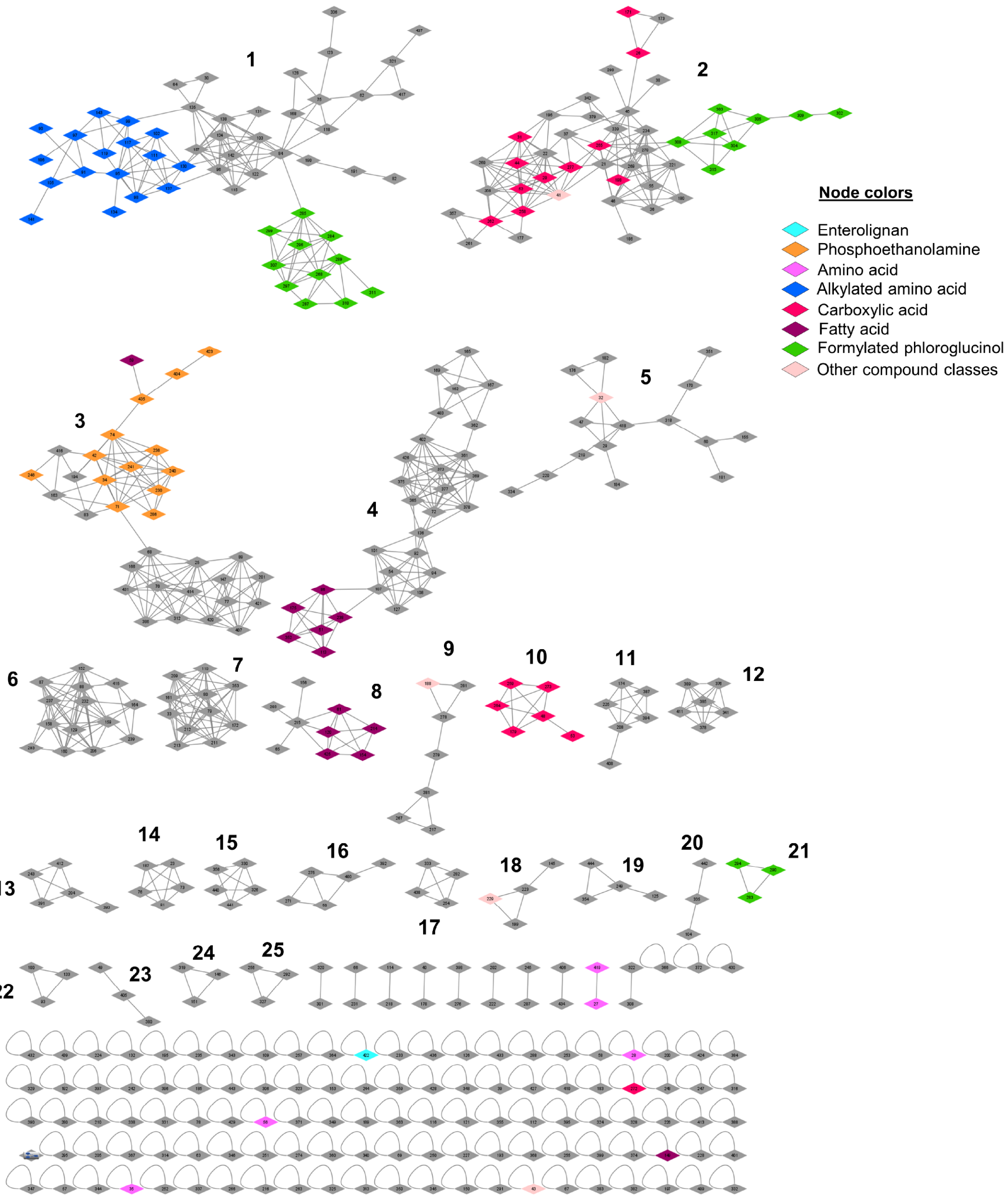

Figure S1. Overview of GNPS networks created for the ESI- features with nodes coloured according to the compound classes of the 94 annotated features (Table S2). Grey nodes represent unannotated features and numbers indicate the network numbers (Table S2).

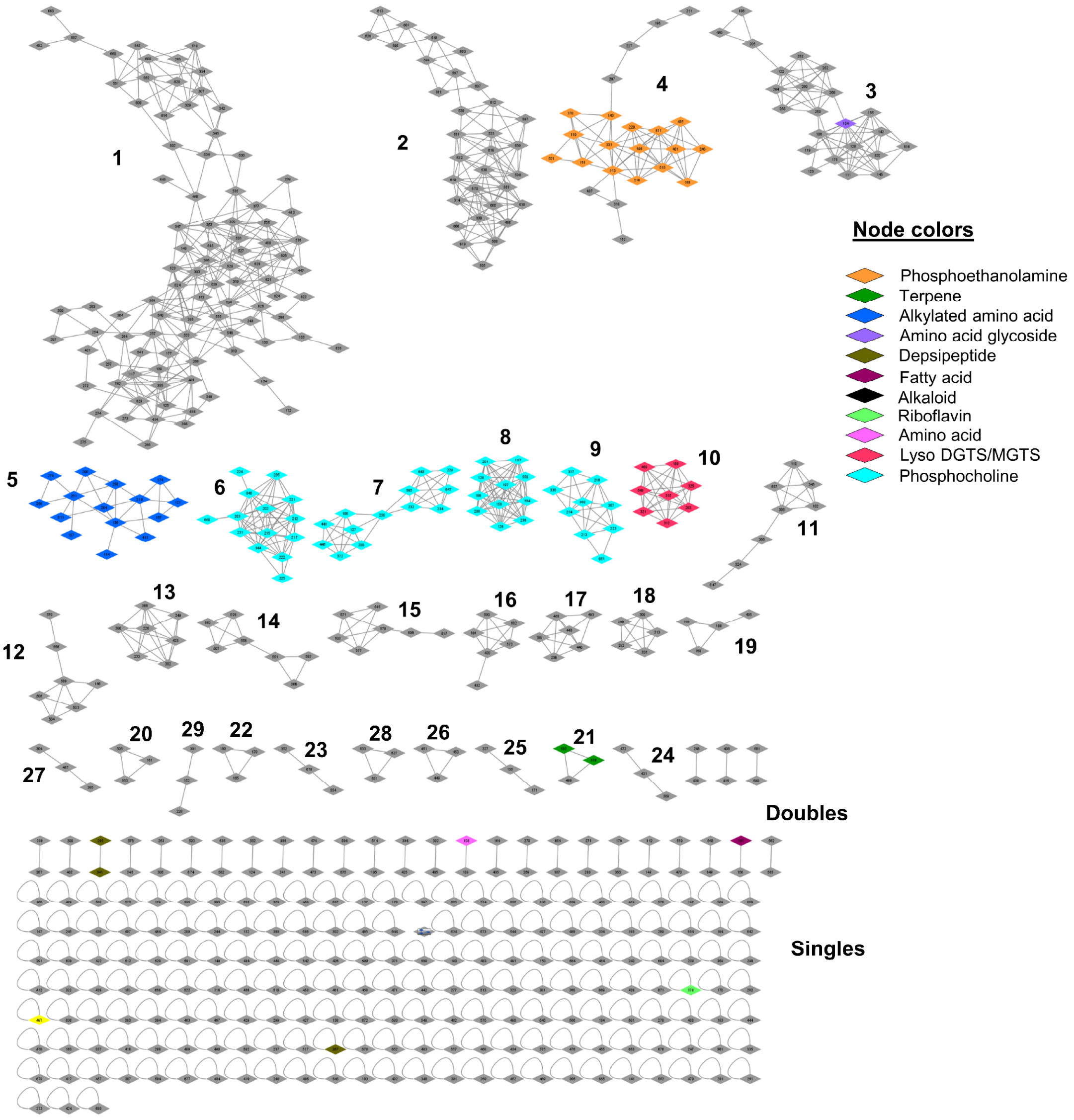

Figure S2. Overview of GNPS networks created for the ESI+ features with nodes coloured according to the compound classes of the 96 annotated features (Table S2). Grey nodes represent unannotated features and numbers indicate the network numbers (Table S2).

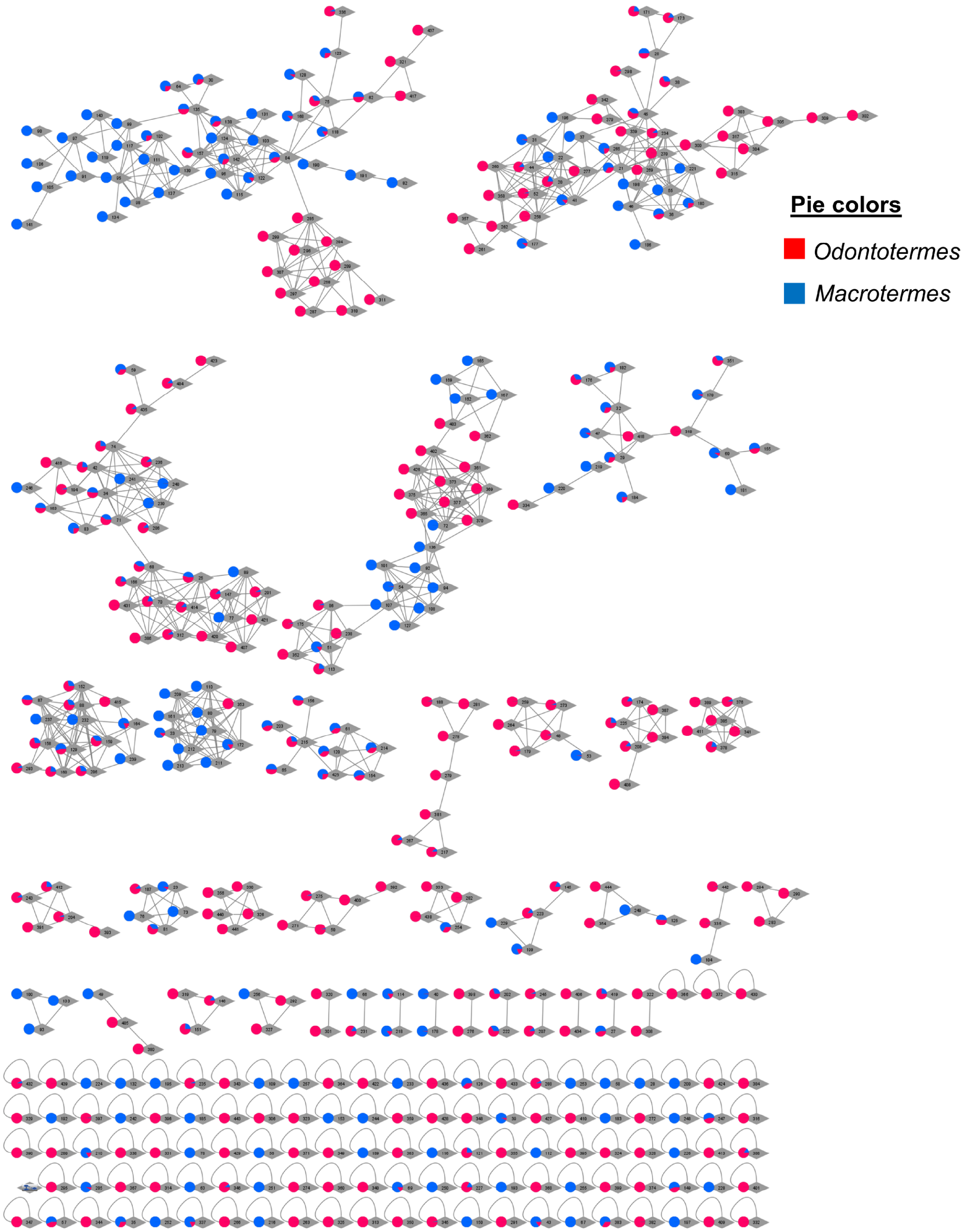

Figure S3. Overview of GNPS networks created for the ESI- features. The coloured pie charts inserted next to the nodes shows the distribution of each feature between termites belonging to each of the two genera, *Odontotermes* and *Macrotermes* (Table S3).

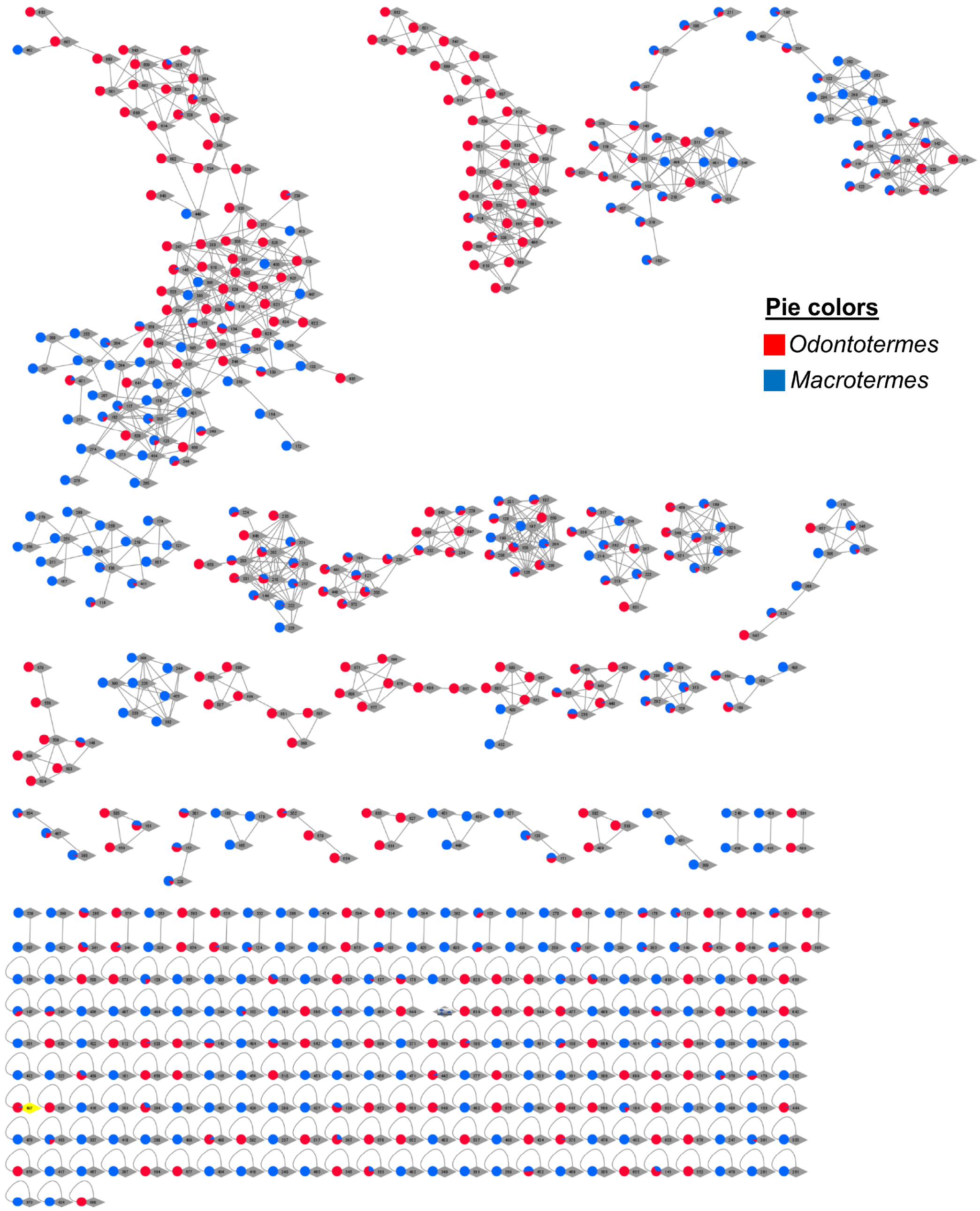

Figure S4. Overview of GNPS networks created for the ESI+ features. The coloured pie charts inserted next to the nodes shows the distribution of each feature between termites belonging to each of the two genera, *Odontotermes* and *Macrotermes* (Table S3).

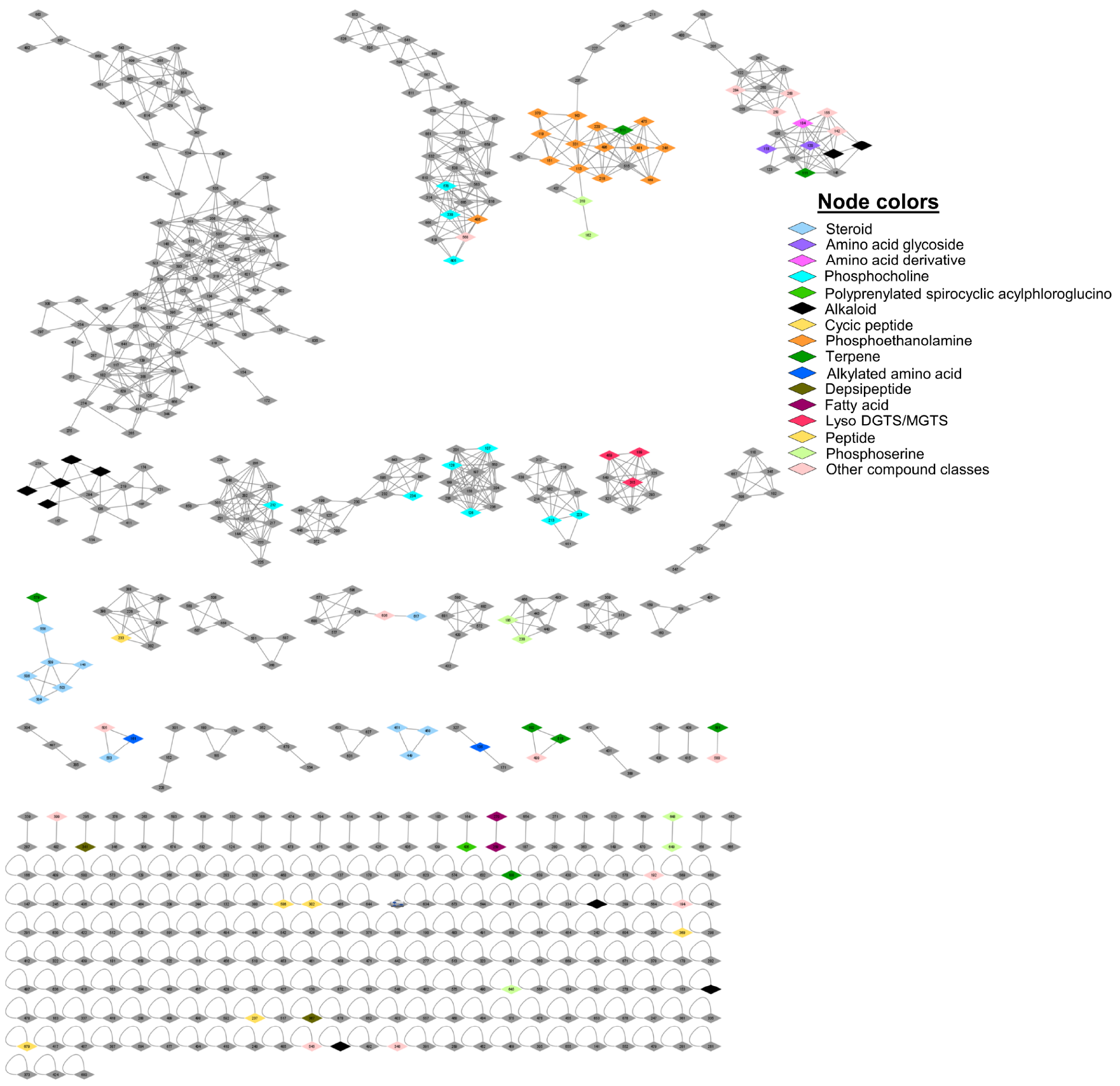

Figure S5. Overview of GNPS networks created for the ESI+ features with nodes coloured according to compound classes suggested from MolDiscovery annotations (Table S2). Grey nodes represent features for which MolDiscovery did not provide an annotation.

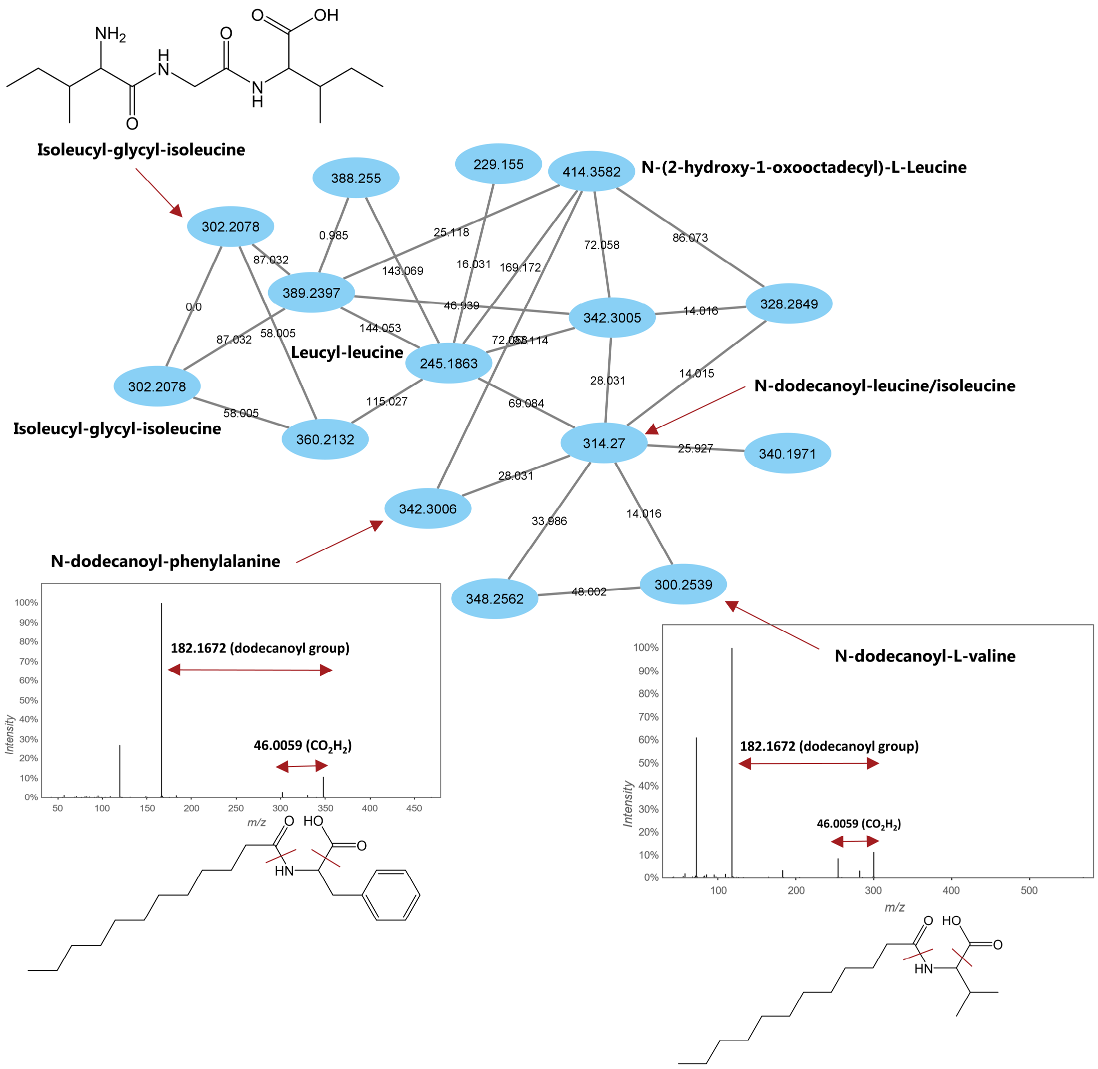

Figure S6. ESI+ GNPS network 5, representing alkylated amino acids and small peptides. The numbers in the nodes are *m*/*z* values of the pre-cursor ions, $[M+H]^+$, whereas numbers along edges are the change in *m*/*z* values between the nodes. Isoleucine-glycyl-isoleucine and leucyl-leucine features were annotated from the NIST14 library. The alkylated amino acids are annotated from our analyses of the MS/MS fragmentation patterns and are also detected with ESI- mode (figure S5). Unannotated nodes were assigned to the compound class, alkylated amino acids/peptides, based on their highly similar fragmentation patterns and retention times (Table S2).

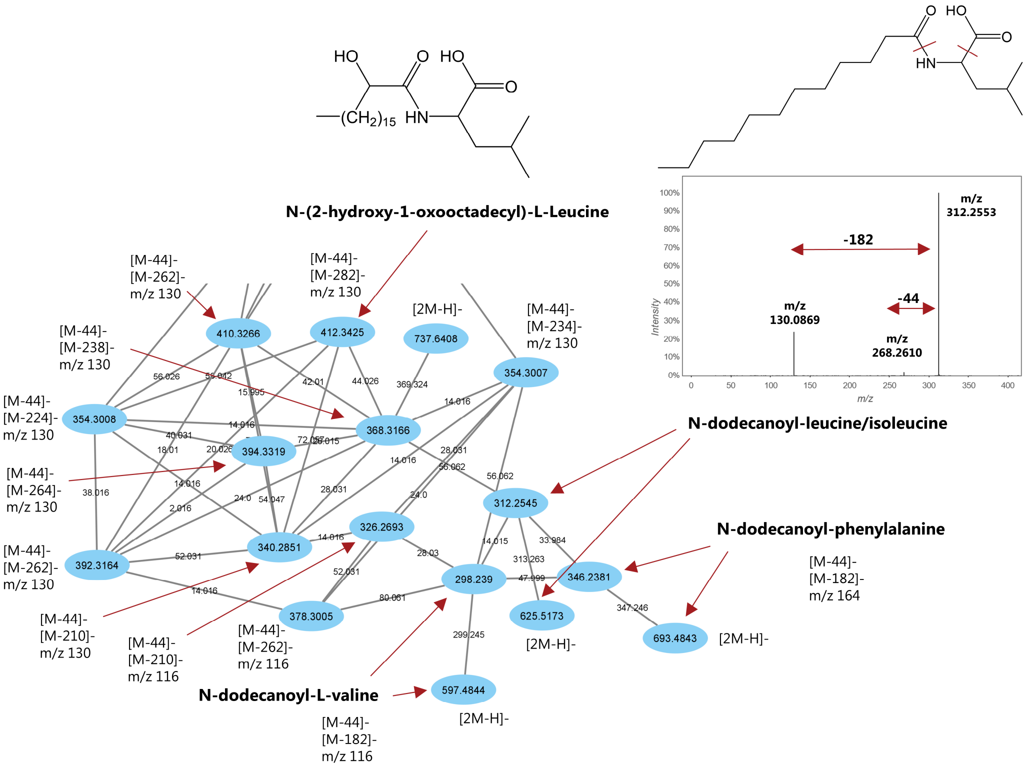

Figure S7. Part of ESI- GNPS network 1, representing alkylated amino acids and potentially small peptides. The numbers in the nodes are *m*/*z* values of the pre-cursor ions, $[M\text{-}H]^-$ or $[2M\text{-}H]^-$, whereas numbers along edges are the change in *m*/*z* values between the nodes. Next to the nodes we display neutral losses (e.g. $[M\text{-}44]^-$ indicate a neutral loss of 44 from $[M\text{-}H]^-$) and *m*/*z* values of significant fragment ions (e.g. *m*/*z* 130). Features annotated as alkylated amino acids were first found in the ESI+ data based on our analyses of the MS/MS fragmentation patterns of the corresponding ESI+ features. The MS/MS fragmentation of the unannotated features of this network share many similarities with the alkylated amino acids with a neutral loss of 44 from [M-H]- representing loss of the carboxylic acid moiety and loss of the sidechain produces the fragment ions corresponding to the parent amino acid. Fragment ions of *m*/*z* 116, 130, and 164 corresponds to valine, leucine/isoleucine and phenylalanine, respectively.

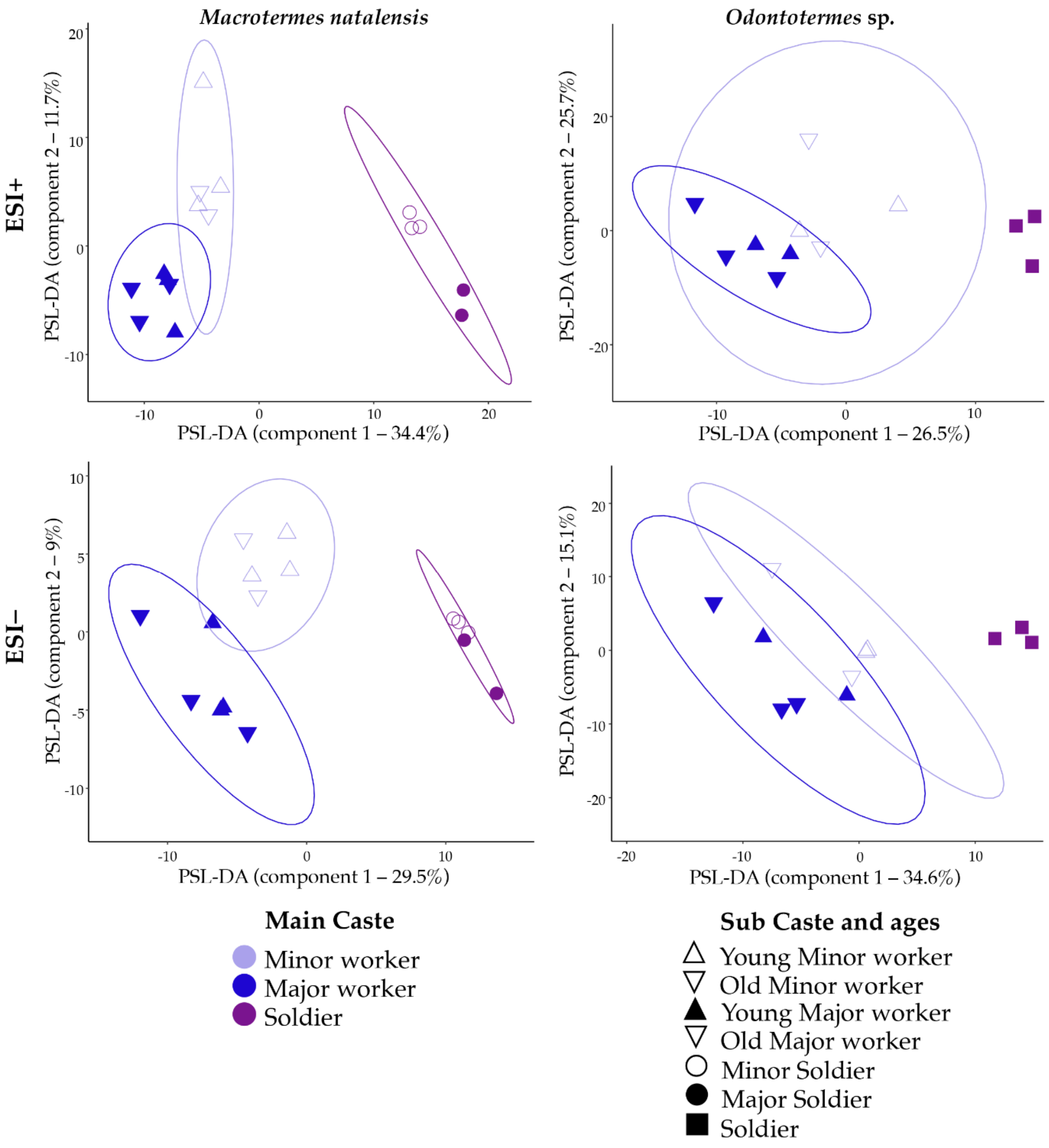

Figure S8. PLS-DA scores plots depicting gut chemical composition of different termite castes in *Macrotermes natalensis* and *Odontotermes* spp. Ellipses represent 95% confident intervals.

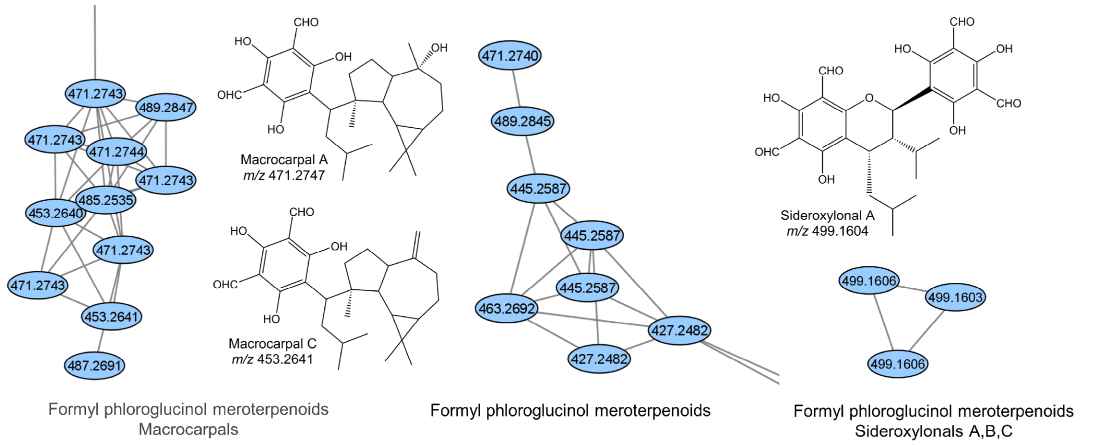

Figure S9. Formylated phloroglucinol compounds (generated in ChemDraw), and their respective small GNPS networks including the *m/z* values of $[M\text{-}H]^-$.

Supplementary Tables with legends

Table S1. List of termite gut samples (separate Excel file)

Table S2. LC-MS/MS feature list. Complete overview of all features with annotations (separate Excel file)

Table S3. List of the normalized LC-MS/MS peak areas for ESI- and ESI+ used for statistical analyses (Separate Excel file)

Table S4. List of significant features from PLS-DAs and ANOVAs (separate Excel file with individual tales in separate sheets as indicated below)

A. Overview of selected top 25/15 VIP features and significant ANOVA features for ESI-

B. Overview of selected top 25/15 VIP features and significant ANOVA features for ESI+

C. Variable importance in projection from PLS-DA of differentially abundant features between termite species in ESI+ mode.

D. Significantly differentially abundant features between termite species in ESI+ mode (one-way ANOVA).

E. Variable importance in projection from PLS-DA of differentially abundant features between termite species in ESI- mode.

F. Significantly differentially abundant features between termite species in ESI- mode (one-way ANOVA).

G. Variable importance in projection from PLS-DA of differentially abundant features between castes in *M. natalensis* in ESI+ mode.

H. Significantly differentially abundant features between castes in *M. natalensis* in ESI+ mode (one-way ANOVA).

I. Variable importance in projection from PLS-DA of differentially abundant features between castes in *M. natalensis* in ESI- mode.

J. Significantly differentially abundant features between castes in *M. natalensis* in ESI- mode (one-way ANOVA).

K. Variable importance in projection from PLS-DA of differentially abundant features between castes in *Odontotermes* sp. in ESI+ mode.

L. Significantly differentially abundant features between castes in *Odontotermes* sp. in ESI+ mode (one-way ANOVA).

M. Variable importance in projection from PLS-DA of differentially abundant features between castes in *Odontotermes* sp. in ESI- mode.

N. Significantly differentially abundant features between castes in *Odontotermes* sp. in ESI- mode (one-way ANOVA).
